# Effects of Radiation Reabsorption on the Flammability Limit and Critical Fuel Concentration of Methane Oxy-Fuel Diffusion Flame

**DOI:** 10.3390/molecules31010124

**Published:** 2025-12-29

**Authors:** Shuochao Wang, Jingfu Wang, Ying Chen, Yi Li, Jiquan Chen, Shun Li, Zewei Yan

**Affiliations:** 1 Beijing Key Laboratory of Heat Transfer and Energy Conversion, Beijing University of Technology, Pingleyuan No. 100, Beijing 100124, China; 2 Beijing Key Laboratory of Control Technology for City Toxic and Combustible Major Hazards, Institute of Urban Safety and Environmental Science, Beijing Academy of Science and Technology, Beijing 100050, China

**Keywords:** oxy-fuel combustion, counterflow diffusion flame, strain rate, radiation reabsorption, statistical narrow-band model, flammable area

## Abstract

This study numerically investigates the critical fuel concentration and flammable regions of methane–air and methane oxy-fuel counterflow diffusion flames. The goal is to determine the effects of strain rate, oxidizer composition, and radiative heat transfer models on flame extinction. Calculations were performed using the counterflow diffusion flame with the adiabatic (ADI), optically thin (OTM), and statistical narrow-band (SNB) radiation models at strain rates of 10 s^−1^, 80 s^−1^, and 200 s^−1^. The key findings are as follows: For methane–air flames, radiation reabsorption has a negligible impact. The flammable region decreases with increasing strain rate (S_Low_ > S_Mid_ > S_High_) across all models. In O_2_/CO_2_ flames, radiation plays a significant role. While the ADI and SNB models maintain the same trend as in air flames, the OTM yields a different order (S_Mid_ > S_High_ > S_Low_). Reducing oxygen concentration increases the critical fuel concentration and shrinks the flammable region. When the oxygen concentration is between 0.35 and 0.40, the combustion characteristics of O_2_/CO_2_ flames resemble those of conventional air flames. In conclusion, this work highlights the critical influence of radiation modeling and oxidizer composition on oxy-fuel flame extinction limits, providing insights for combustion system design under CO_2_ dilution.

## 1. Introduction

Reducing pollutant emissions is a primary goal in the field of combustion [1]. Additionally, the reduction of NOx and CO_2_ in combustion products has consistently been a major research focus. When a fuel is a common hydrocarbon, which employs air as the oxidant, it produces combustion products that include not only CO_2_ and H_2_O but also nitrogen oxides and other gases, significantly complicating carbon capture. To address this, a technology combining pure oxygen combustion with carbon capture and storage (CCS), which can substantially reduce CO_2_ emissions, has been proposed and developed [2,3,4,5,6]. However, due to the distinct physical and chemical properties of N_2_ and CO_2_, oxy-fuel combustion flames exhibit notable differences compared with conventional air–fuel flames [7,8,9,10,11], particularly in terms of fuel conversion efficiency and flame temperature [9]. Therefore, to achieve the industrial application of this combustion technology, it is necessary to deeply explore its fundamental combustion characteristics, especially the unique radiative heat transfer problems caused by using CO_2_ as the main diluent.

Previous research on O_2_/CO_2_ flames has primarily focused on fundamental combustion characteristics such as flame temperature [12,13,14,15,16,17] and laminar flame speed [18,19,20,21,22]. Through experimental and numerical methods, respectively, Wu [14] and Zhao [17] investigated the effects of adding N_2_ and CO_2_ as diluents to fuel. They found that at the same dilution concentration, CO_2_ led to a decrease in flame temperature and a narrowing of the flammable range compared with N_2_ due to its combined physical and chemical effects. A. A. Konnov et al. [18] employed the heat flux method to measure the combustion of CH_4_ in an O_2_/CO_2_ atmosphere. The results demonstrated that laminar flame speed increases with rising oxygen concentrations, while it first increases and then decreases with increasing equivalence ratios. Hu [21,22] measured the laminar burning velocity of O_2_/CO_2_ flames using a Bunsen burner under both ambient and elevated temperature oxidizer conditions. The results demonstrated that the addition of CO_2_ suppresses the laminar burning velocity at ambient temperature, which is attributed to its thermal, radiative, and chemical effects. As the oxidizer temperature increases, the elevated temperature of the unburned gas raises the adiabatic flame temperature, thereby accelerating the reaction rate of the mixture and enhancing the laminar burning velocity.

Flame stability is one of the most critical parameters in the combustion process [23,24,25], providing essential theoretical support for the design of industrial burners. The study of flame stability primarily focuses on its extinction limits and lean flammability limit. Research on the stretch extinction limit of O_2_/CO_2_ flames is relatively well-established [26,27,28,29]. Li [27] and Zhang [29] numerically determined the stretch extinction limits of CH_4_/CO_2_ versus O_2_/CO_2_ counterflow diffusion flames under high-temperature oxidizer and high-pressure conditions, respectively. Their results demonstrate that an O_2_/CO_2_ flame with an oxygen concentration of 0.35 exhibits a stretch extinction limit comparable to that of an air flame. Increasing the oxidizer temperature or system pressure significantly enhances the extinction limit. Furthermore, radiative effects on extinction become negligible when a high-temperature oxidizer is used. By decoupling the chemical and radiative effects of CO_2_, Kim [28] observed that radiative heat loss plays a significant role at low strain rates, while the chemical effect of CO_2_ substantially reduces the critical diluent mole fraction at extinction. In oxygen-enriched combustion systems involving high concentrations of carbon dioxide, the influence of radiative heat transfer mechanisms on stability is particularly crucial, as CO_2_ has distinct radiative characteristics from N_2_.

Radiation reabsorption is a significant heat transfer mechanism in combustion processes. Considerable progress has been made in recent studies on the effects of radiation reabsorption in flame propagation. Zheng et al. [30] investigated the influence of radiation reabsorption on the laminar burning velocity of NH_3_/H_2_/air flames. Their results indicate that radiation reabsorption not only directly affects radiative heat loss but also preheats unburned mixtures, thereby enhancing flame propagation. For conventional stretched counterflow flames, radiation reabsorption significantly affects the critical fuel concentration at the extinction limit. Maruta et al. [26] compared and analyzed the extinction limits of CH_4_/CO_2_ and O_2_/CO_2_ diffusion flames under high pressure through experimental and numerical methods. Their results demonstrate that the predictions of the statistical narrow-band (SNB) model agree more closely with experimental measurements, highlighting the considerable importance of radiation reabsorption under elevated pressure conditions. Okuno et al. [31] conducted microgravity experiments on CH_4_/O_2_/CO_2_ counterflow premixed flames and performed complementary simulations using the SNB model. For the first time, flame bifurcation was observed in the numerical results, indicating that the extinction curve of a counterflow premixed flame exhibits a distinct “G” shape. Li et al. [32] measured the extinction limits of oxy-fuel flames under both normal gravity and microgravity conditions, and they numerically obtained a C-shaped extinction curve for the counterflow diffusion flame. The results showed that when radiation reabsorption was considered, the calculated lean flammability limit agreed well with experimental data, whereas predictions from an optically thin model (OTM) were slightly higher.

Although the above-mentioned research has made significant progress in revealing the influence of radiation reabsorption on the macroscopic characteristics of flames (such as flameout limit), a key quantitative understanding for engineering applications is still lacking: how radiation reabsorption systematically affects two core engineering parameters that determine flame stability—critical fuel concentration and combustible zone. Specifically, under a wide range of strain rates and oxygen concentrations, the quantitative correction rules for the critical fuel concentration of oxy-fuel diffusion flames by reabsorption have not yet been clarified. The changes in the flammable zone caused by it and the differences from traditional air flames also lack systematic comparison. This lack of quantitative cognition directly restricts the effective application of high-precision radiation models in the stability design and optimization of oxygen-enriched burners. To facilitate the broader application of oxy-fuel combustion technology in industrial burners [33], this study aimed to systematically quantify the quantitative effects of radiation reabsorption on the flame temperature, critical fuel concentration and combustible zone of methane oxygen-enriched hedging diffusion flames through numerical simulation. We adopted adiabatic models, optical thin models and statistical narrowband models for comparison, focusing on analyzing the variation laws of flame stability under different strain rates and oxygen concentrations, and we compared the results with methane–air flames to clarify the key role of radiation reabsorption in oxy-fuel combustion.

## 2. Results and Discussion

### 2.1. Methane–Air Counterflow Diffusion Flame

#### 2.1.1. Overall Trends of Flame Temperature Versus Strain Rate Under Different Radiation Models

**Figure 1:**
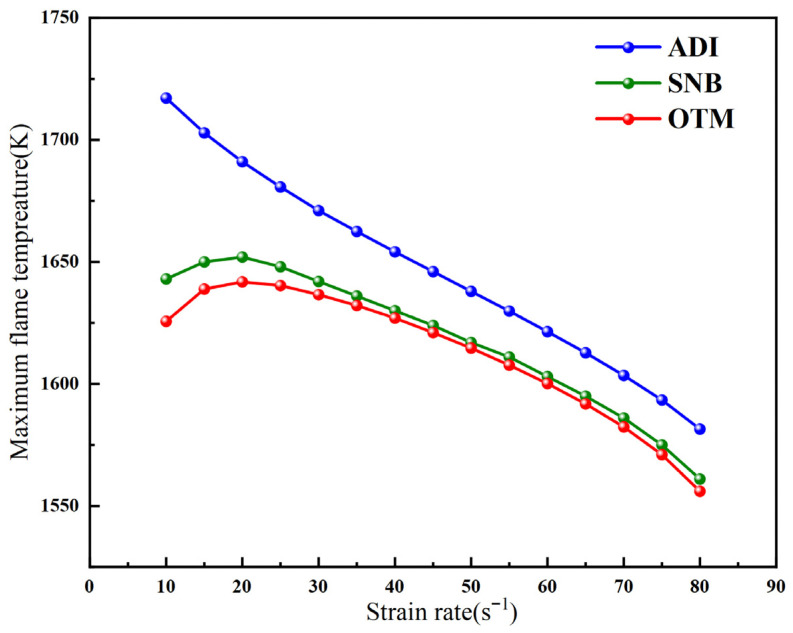
The variation of the maximum flame temperature and strain rate of the methane–air flame under different radiation models.

Figure 1 shows the variation of flame temperature with strain rate for a methane–air diffusion flame at X_F_ = 0.2 under different radiation models. As can be observed, for the ADI model, the maximum flame temperature increases as the strain rate decreases [34]. When radiative heat loss is neglected, a lower strain rate allows for longer gas residence time, leading to more complete combustion and, consequently, a higher flame temperature. In contrast, the results obtained from the SNB and OTM simulations show that the maximum flame temperature initially increases and then decreases with an increasing strain rate. This non-monotonic trend is attributed to the competing effects between gas residence time and radiative heat loss. Furthermore, the results from both radiation models indicate that the difference in maximum flame temperature is negligible at high strain rates, while the effect of radiation reabsorption only becomes significant at low strain rates.

**Figure 2:**
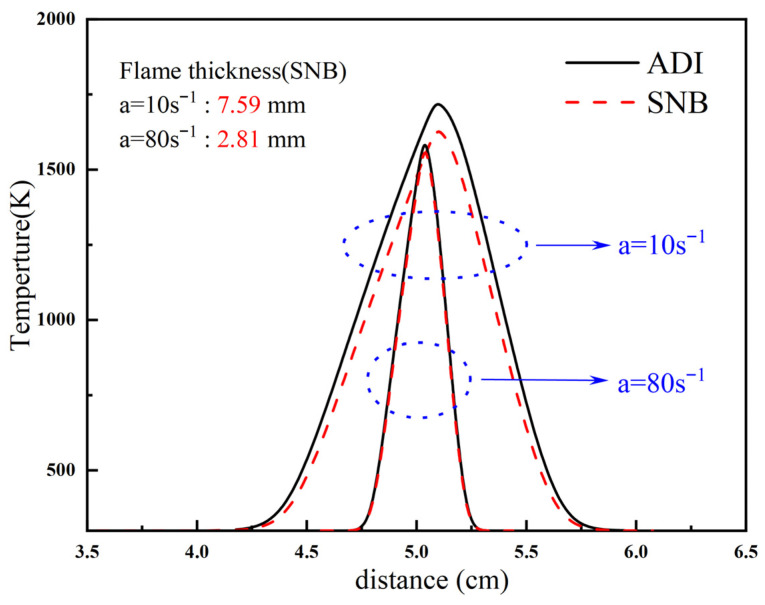
Flame temperature distribution diagrams of a = 10 s^−1^ and a = 80 s^−1^ under the ADI and SNB models.

Figure 2 presents the flame temperature profiles calculated by the ADI and SNB models at strain rates of a = 10 s^−1^ and a = 80 s^−1^. As clearly shown, at a = 10 s^−1^, the maximum temperature difference between the ADI and SNB model results is ΔT = 74.127 K. The flame thickness, calculated using the full width at half maximum (FWHM) method proposed by Sung et al. [35], is d = 7.59 mm at this strain rate, indicating significant radiative effects. In contrast, at a = 80 s^−1^, the two temperature profiles nearly overlap, with a maximum temperature difference of only ΔT = 20.539 K and a flame thickness of d = 2.81 mm, suggesting negligible radiative heat loss. It is evident that flame thickness decreases with an increasing strain rate, while radiative heat loss becomes more pronounced as the flame thickens. Consequently, at low strain rates, the influence of radiative heat loss dominates the effect of gaseous fuel residence time. At high strain rates, however, the shorter residence time becomes the governing factor and the impact of radiative heat loss on the overall reaction can be considered negligible.

**Figure 3:**
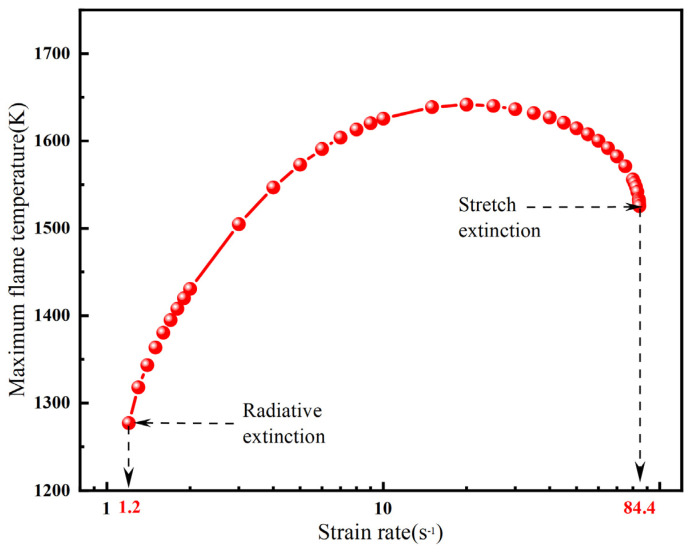
The overall trend of the maximum temperature of the methane–air flame with X_F_ = 0.2 varying strain rate (OTM).

Figure 3 illustrates the overall trend of maximum temperature versus strain rate for a methane–air flame at X_F_ = 0.2 under the OTM. As previously discussed, as the strain rate continues to decrease, the increasing flame thickness leads to greater radiative heat loss. Eventually, the heat loss due to radiation exceeds the heat release from chemical reactions, resulting in flame extinction. This extinction limit is termed the radiative extinction, which occurs at a = 1.2 s^−1^ and an extinction temperature of T_max_ = 1277.04 K. Conversely, as the strain rate increases, the residence time of the gaseous fuel becomes shorter than the characteristic chemical reaction time, also leading to extinction. This limit is referred to as the stretch extinction, occurring at a = 84.4 s^−1^ and T_max_ = 1525.585 K. It is important to note that the radiation extinction at low strain rates only manifests when radiative heat loss is considered. In contrast, the adiabatic (ADI) model only predicts the stretch extinction at high strain rates.

#### 2.1.2. Critical Fuel Concentration and Flammable Area Under Different Radiation Models

It has been well established that the extinction limits of methane–air counterflow diffusion flames exhibit a characteristic C-shaped curve [36]. This implies the existence of a critical fuel concentration, below which the flame will extinguish regardless of the strain rate. Li et al. [32] experimentally identified a lean fuel limit of X_F_ = 0.15 for methane–air flames under microgravity conditions, and their numerical calculations using the OTM predicted this critical value to be X_F_ = 0.13. However, no studies to date have systematically investigated the critical fuel concentration and the corresponding flammable area of flames across varying strain rates.

**Figure 4:**
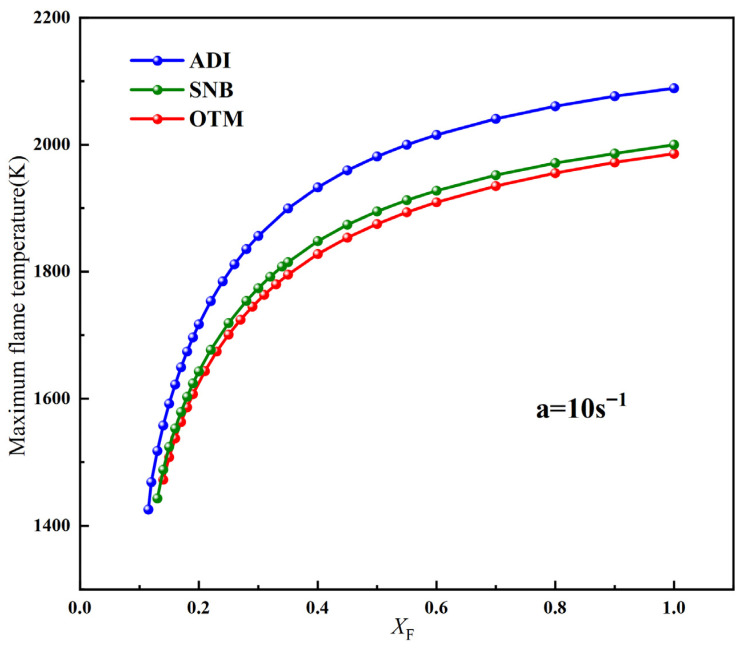
The variation of the maximum flame temperature with X_F_ at a = 10 s^−1^ for different radiation models.

Figure 4 shows the variation of the maximum flame temperature with fuel concentration for an air flame at a = 10 s^−1^, as calculated using the ADI, OTM, and SNB radiation models. It can be observed that the trend of the maximum flame temperature across different methane concentrations computed by the SNB model shows little difference from that of the OTM. When the methane concentration on the fuel side is high, the maximum flame temperature calculated by all three models is largely insensitive to changes in fuel concentration. However, when X_F_ < 0.3, the maximum flame temperature significantly changes with decreasing fuel concentration until extinction occurs. In this study, the critical fuel concentration is clearly defined as the minimum molar fraction (X_F_)_min_ of methane in the fuel flow that can maintain a stable combustion of the counterflow diffusion flame under the given strain rate a, oxidant oxygen concentration X_O_, and radiation model. When the fuel concentration drops below this critical value, the flame will extinguish no matter how the other conditions are optimized. Therefore, for different radiation models, there exists a specific (X_F_)_min_ at a fixed strain rate. If X_F_ < (X_F_)_min_, the flame will be extinguished.

Using the same method, we calculated the critical fuel concentration of methane–air diffusion flames under conditions of a = 80 s^−1^ and a = 200 s^−1^ while employing the ADI, OTM, and SNB radiation models. The results are summarized in Table 1.

**Figure 5:**
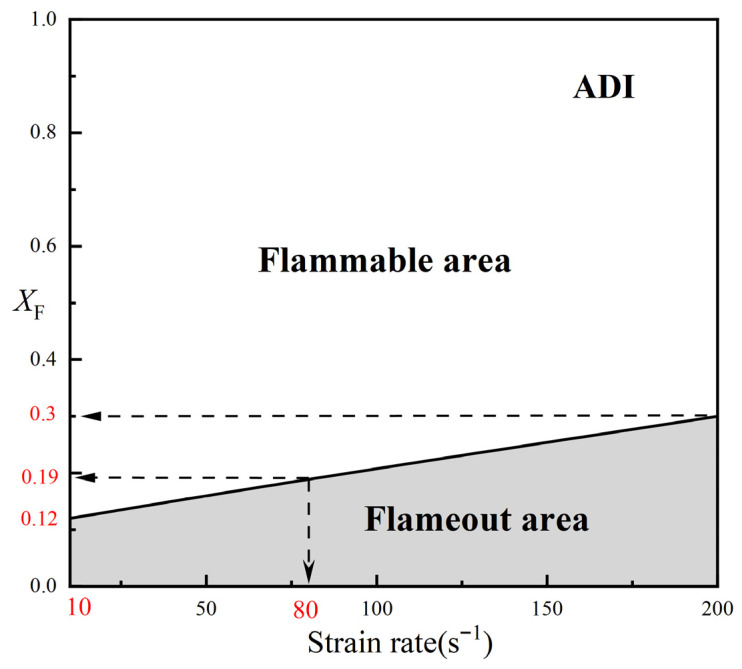
Diagram of the flammable area of the methane–air diffusion flame at different strain rates (ADI).

The calculated fuel concentration limits under ADI conditions at various strain rates were plotted as a continuous curve, as shown in Figure 5. The upper portion of the curve, where X_F_ > (X_F_)_min_, corresponds to the flammable area (indicated by the blank region in the figure). Conversely, when the fuel concentration falls below the critical limit, it is considered the flameout area (shown as the shaded region). Under adiabatic conditions, lower strain rates result in higher flame temperatures and a broader flammable range.

**Figure 6:**
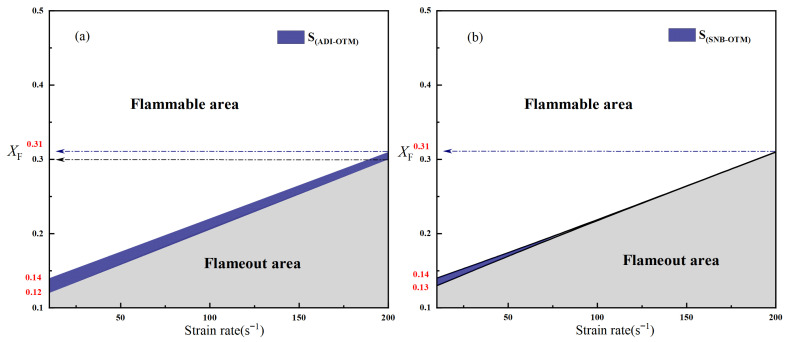
Comparison of the flammable area of methane–air diffusion flames under different radiation models (**a**) Calculated by the ADI and OTM models. (**b**) Calculated by the SNB and OTM models.

Under the OTM, the calculated minimum fuel concentrations are (X_F_)_min_ = 0.14 at the lower extinction limit, (X_F_)_min_ = 0.20 at a = 80 s^−1^, and (X_F_)_min_ = 0.31 at a = 200 s^−1^. A comparison of the flammable ranges between the OTM and ADI models is presented in Figure 6a. The blank area indicates the region where the flame is flammable under both models. The shaded region represents the flameout zone, while the blue area denotes the difference in flammable area between the ADI and OTM models’ calculated results. As can be clearly observed, the difference in area is more pronounced at low strain rates, while the discrepancy in the flammable area becomes smaller at medium and high strain rates. Considering the gas radiation reabsorption effect, the SNB model yields the following calculated results: (X_F_)_min_ = 0.13 at a = 10 s^−1^, (X_F_)_min_ = 0.20 at a = 80 s^−1^, and (X_F_)_min_ = 0.31 at a= 200 s^−1^. A comparison between the SNB and OTM calculations is presented in Figure 6b, aiming to reveal the influence of the reabsorption effect on the critical fuel concentration and flammable region of methane–air flames. It can be clearly observed that the two curves essentially coincide at medium and high strain rates, with deviations only occurring at low strain rates. This phenomenon can be explained as follows: at high strain rates, the flame thickness becomes sufficiently small that radiation reabsorption effects can be neglected, making the OTM suitable for calculating methane–air diffusion flames under such conditions. In contrast, at low strain rates, the use of the SNB model is more appropriate due to the greater reabsorption influence. Although flames at low strain rates are more sensitive to radiative heat loss, the flammable region of methane–air flames remains the largest under any radiation model at these lower strain rates.

In summary, the flammable areas under low, medium, and high strain rates satisfy the relationship: S_Low_ > S_Mid_ > S_High_. Meanwhile, the flammable areas calculated with different radiation models follow the order: S_ADI_ > S_SNB_ > S_OTM_. The influence of radiation reabsorption on methane–air diffusion flames is relatively small.

### 2.2. Methane Oxy-Fuel Counterflow Diffusion Flame

#### 2.2.1. Overall Trends of Flame Temperature Versus Strain Rate Under Different Radiation Models

**Figure 7:**
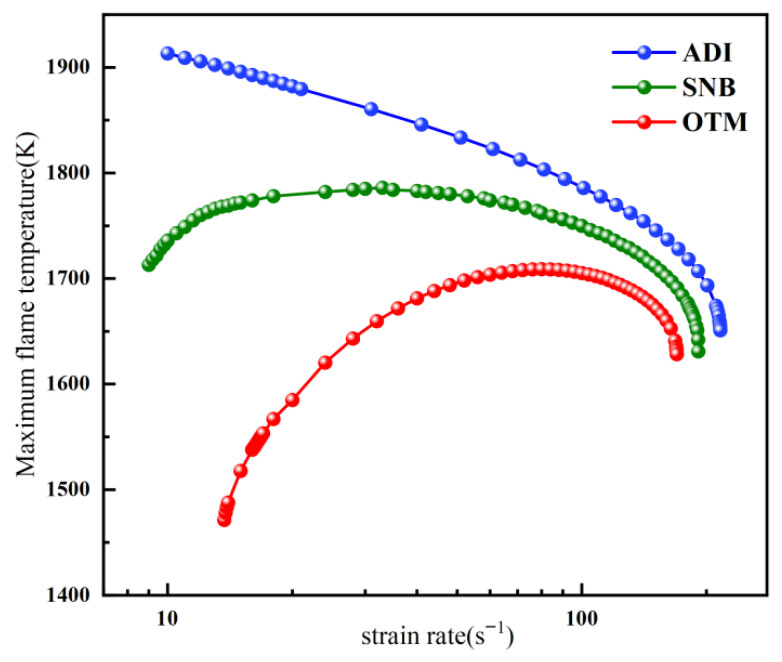
The overall trend of the maximum temperature of methane oxy-fuel flame varying strain rate (X_F_ = 0.3, X_O_ = 0.35) under different radiation models.

Since CO_2_ is a strongly radiating gas, its radiative effects had to be considered in the calculations. Accordingly, the complete variation of flame temperature with strain rate was computed for a diffusion flame with X_F_ = 0.3 and X_O_ = 0.35 using different radiation models, as shown in Figure 7. The variation of maximum flame temperature with strain rate under different radiation models is generally consistent with that of methane–air flames. Under both the OTM and SNB models, stretch extinction and radiation extinction limits are observed. In contrast, only a stretch extinction limit exists under the ADI model. However, the radiative effect in O_2_/CO_2_ flames is significantly stronger than in air flames. Moreover, when radiation reabsorption is considered, the maximum flame temperature occurs at a lower strain rate compared with the OTM, which neglects reabsorption. This can be primarily attributed to the fact that radiation reabsorption allows part of the radiatively lost energy to be reabsorbed within the flame itself rather than being entirely lost to the external environment. An increase in strain rate leads to a thinner flame, which significantly weakens radiation reabsorption and results in a rapid rise in net radiative heat loss. Concurrently, the reduced residence time of the flame collectively contributes to an earlier occurrence of the temperature peak. A comparison between the SNB and OTM model curves reveals that the discrepancy in the calculated maximum flame temperature between the two models decreases with increasing strain rate. This indicates that different radiation models have an insignificant influence on the stretch extinction limit but exert a notable effect on the radiation extinction limit.

**Figure 8:**
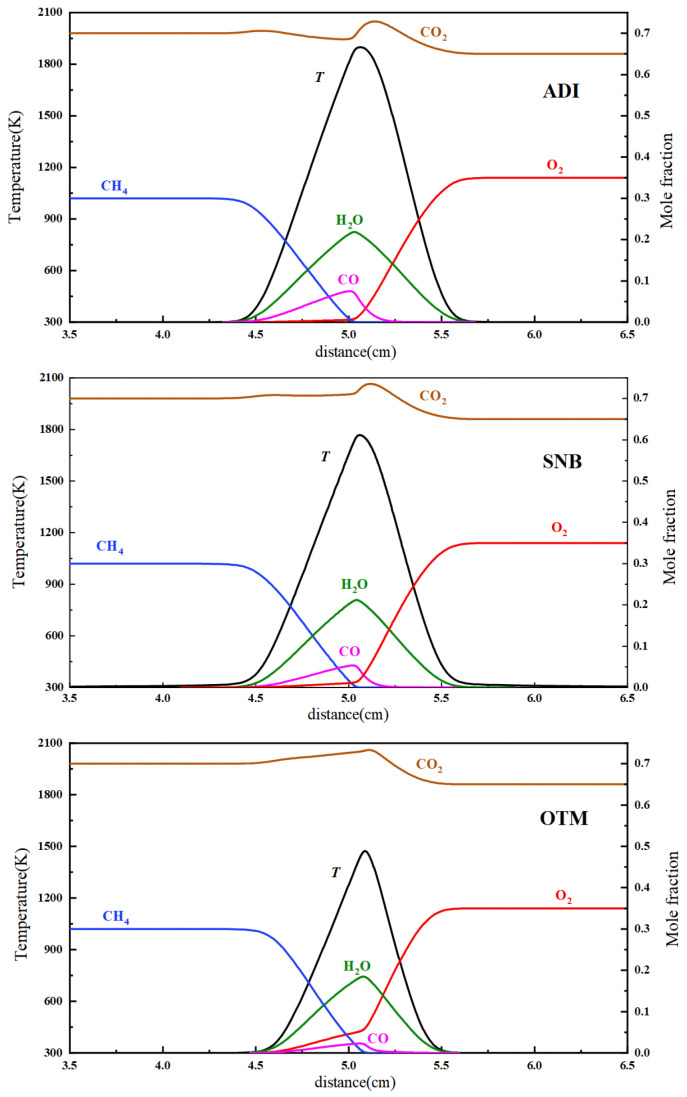
Computational flame structures of the methane oxy-fuel flame (X_F_ = 0.3, X_O_ = 0.35, a = 13.7 s^−1^) calculated using different radiation models.

Figure 8 shows the temperature and major species concentration distributions calculated using different radiation models at X_F_ = 0.3, X_O_ = 0.35, and a = 13.7 s^−1^. The results indicate that the overall flame structure is similar to that of a conventional methane–air flame, except for a higher CO_2_ concentration. Additionally, the concentration distributions of the major species are similar across the three radiation models, while significant differences are observed in the flame temperature. The peak flame temperatures under these conditions are 1899 K for the ADI model, 1768 K for the SNB model, and 1473 K for the OTM. Unlike conventional air flames, O_2_/CO_2_ flames exhibit greater sensitivity to radiation effects in their temperature distribution at low strain rates.

#### 2.2.2. Critical Fuel Concentration and Flammable Regions Under Different Radiation Models

**Figure 9:**
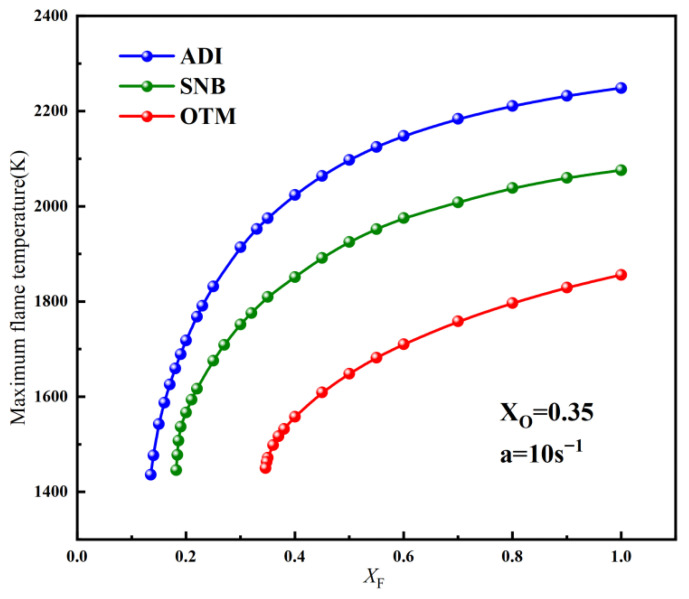
The variation of the maximum flame temperature with X_F_ at a = 10 s^−1^ for different radiation models of methane oxy-fuel.

Calculations for conventional air flames indicate that radiation has a relatively small effect on the critical fuel concentration and flammable area. In contrast, O_2_/CO_2_ flames are more sensitive to radiative heat loss, necessitating an investigation into their critical fuel concentrations and flammable area. Unlike the approach used for air flames, the critical methane concentration on the fuel side was determined at a fixed strain rate by progressively varying the oxygen concentration on the oxidizer side. Initially, the variation of maximum flame temperature with different methane concentrations on the fuel side was computed at a = 10 s^−1^ and X_O_ = 0.35 using different radiation models, as shown in Figure 9.

Unlike conventional air flames, a significant discrepancy in the maximum flame temperature is observed between the SNB and OTM models, indicating a stronger influence of gas reabsorption on flame temperature. Similarly, as the methane concentration on the fuel side decreases, the maximum flame temperature drops sharply until extinction occurs. Hence, the minimum methane concentration required to sustain combustion is defined as (X_F_)_min_. The calculated values of (X_F_)_min_ are 0.14 for the ADI model, 0.19 for the SNB model, and 0.35 for the OTM. Significant differences in (X_F_)_min_ among the three radiation models can be observed, indicating that O_2_/CO_2_ flames are more sensitive to radiative heat loss.

**Figure 10:**
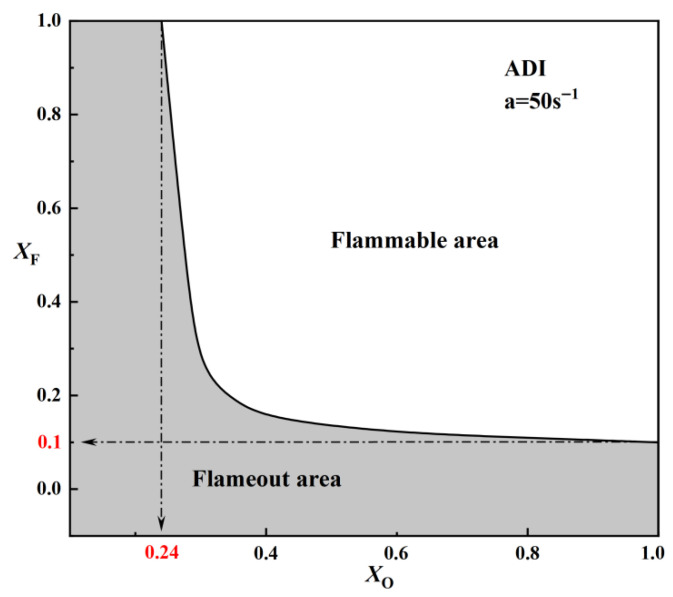
Diagram of the combustible area of methane oxy-fuel flame under different oxygen concentrations (ADI, a = 50 s^−1^).

Under the ADI model, at a strain rate of a = 50 s^−1^, the critical fuel concentration (X_F_)_min_ was calculated for X_O_ = 1, 0.8, 0.6, 0.4, 0.35, and 0.3. Additionally, the critical oxygen concentration (X_O_)_min_ was determined under the condition X_F_ = 1. The results show that at a fixed strain rate, (X_F_)_min_ increases as the oxygen concentration decreases. When X_O_ = 1, (X_F_)_min_ is 0.1. This indicates that at a = 50 s^−1^, the flame will be extinguished under any oxygen concentration if the methane concentration on the fuel side is below 0.1. When X_F_ = 1, the calculated (X_O_)_min_ is 0.24. This means that at a = 50 s^−1^, the flame will be extinguished under any fuel concentration if the oxygen concentration on the oxidizer side falls below 0.24. The (X_F_)_min_ values calculated at different oxygen concentrations were plotted as a curve to visualize the flammable area, as shown in Figure 10. The curve divides the entire region into two parts: when X_F_ > (X_F_)_min_, the flame is flammable, corresponding to the blank area in the figure, which represents the flammable region at a = 50 s^−1^. When X_F_ > (X_F_)_min_, the flame is extinguished, as represented by the shaded area in the figure, which corresponds to the flameout area at a = 50 s^−1^.

To investigate the flammable regions under different strain limits for the three radiation models, we calculated the critical fuel concentrations at various oxygen concentrations. The strain rates were selected as a = 10 s^−1^ for the low range, a = 80 s^−1^ for the medium range, and a = 200 s^−1^ for the high range. First, the (X_F_)_min_ values at different oxygen concentrations were calculated using the three radiation models, as summarized in Table 2. The results show that the variation trend of the critical fuel concentration obtained with the ADI and SNB models is similar to that of air flames: as the strain rate decreases, the critical fuel concentration also decreases. This implies that a flame can be sustained at lower methane concentrations, resulting in an expanded flammable area. In contrast, the OTM results indicate that as the strain rate decreases, the critical fuel concentration first decreases and then increases, with the (X_F_)_min_ at low strain rates being higher than that at high strain rates. The critical oxygen concentration (X_O_)_min_ was also calculated for pure methane on the fuel side under different radiation models and strain rates, as shown in Table 3. It can be observed that the values of (X_O_)_min_ obtained with the ADI and SNB models show only minor differences at low strain rates. In contrast, the (X_O_)_min_ values computed with the OTM exhibit a trend similar to that of (X_F_)_min_, significantly deviating from the adiabatic flame results at low strain rates.

**Figure 11:**
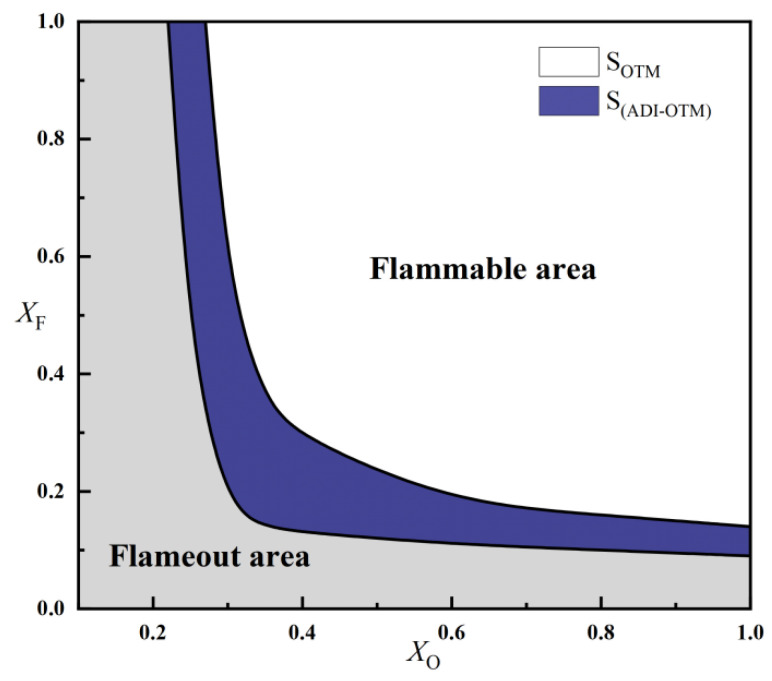
Comparison of the flammable area of methane oxy-fuel diffusion flames under ADI and OTM models (a = 10 s^−1^).

Based on the calculated results, the differences between (X_O_)_min_ and (X_F_)_min_ at a = 80 s^−1^ and a = 200 s^−1^ are relatively small. Therefore, the focus remains on examining the flammable area of methane oxy-fuel diffusion flames under different radiation models at a = 10 s^−1^. Figure 11 shows the flammable area calculated using the ADI and OTM models at a = 10 s^−1^. The results indicate that S_ADI_ > S_OTM_, with a significantly larger discrepancy between the two models compared with that observed in methane–air flames. Notably, the difference in predicted flammable areas is most pronounced at medium oxygen concentrations (X_O_ = 0.35–0.5). This occurs because at high oxygen concentrations, the flame temperature is extremely high and radiative heat loss becomes significant. However, the discrepancy between the ADI and OTM model predictions in this regime is primarily dominated by high-temperature chemical reactions. As the oxygen concentration decreases, the flame temperature continues to drop, reducing the effect of radiation and thus narrowing the gap between the ADI and OTM model results. It can be inferred that at medium oxygen concentrations, an optimal balance is reached between radiative loss and chemical reaction rates. Under these conditions, the OTM requires a substantially higher fuel concentration to compensate for radiative losses.

**Figure 12:**
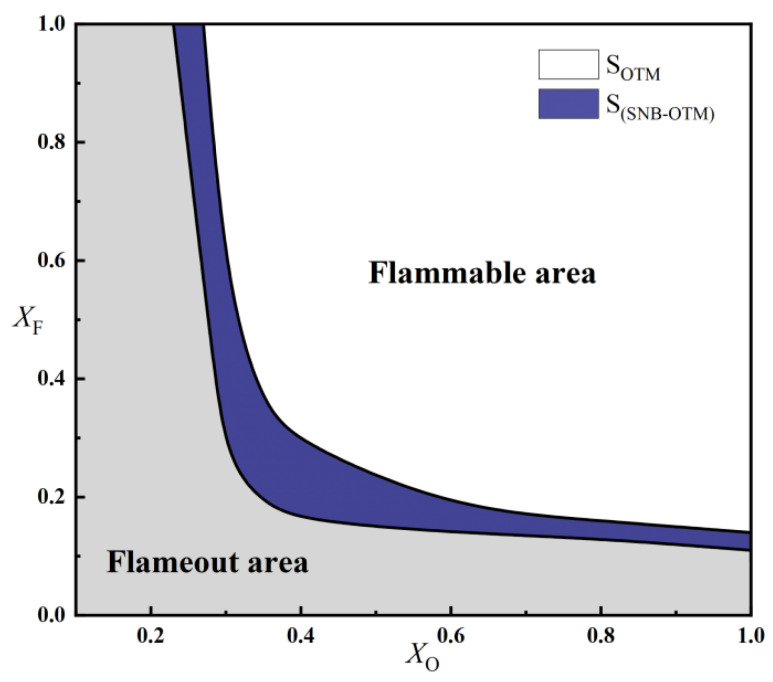
Comparison of the flammable area of methane oxy-fuel diffusion flames under SNB and OTM models (a = 10 s^−1^).

To investigate the effect of radiation reabsorption on the flammable region, the flammable areas calculated using the SNB and OTM models at a = 10 s^−1^ are compared in Figure 12. The results show that S_SNB_ > S_OTM_, indicating that radiation reabsorption has a greater influence on O_2_/CO_2_ flames than on air flames. Similarly, the difference in flammable area between the two models is largest at medium oxygen concentrations (X_O_ = 0.35–0.5). This is primarily because the radiation reabsorption effect is particularly significant under conditions of high temperature and thick planar flames. When X_O_ > 0.5, the flame temperature remains relatively stable, the proportion of radiative heat loss decreases, and radiation reabsorption weakens, leading to a reduced discrepancy in the flammable area between the SNB and OTM models. When X_O_ < 0.35, the overall flame temperature decreases, which also decreases the radiation reabsorption effect.

### 2.3. A Comparison of Critical Fuel Concentration Between Air Flames and Oxy-Fuel Flames

**Figure 13:**
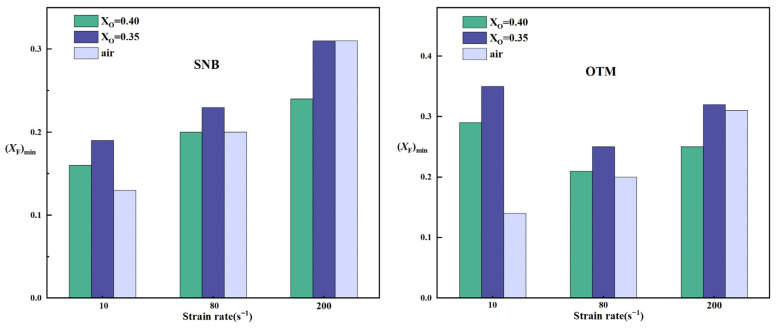
Comparison of critical fuel concentrations between air flame and oxy-fuel flame with X_O_ = 0.35 and 0.4 under different radiation models.

We compared the critical fuel concentrations of methane–air and methane oxy-fuel flames calculated under different models, as shown in Figure 13.

When using the OTM model, the (X_F_)_min_ calculated for air flames at high strain rates falls within the range of X_O_ = 0.35–0.4 for oxy-fuel flames. In contrast, at low strain rates, the critical fuel concentration of air flames is significantly lower than that of O_2_/CO_2_ flames at X_O_ = 0.35–0.4.

When radiation reabsorption is considered, the (X_F_)_min_ of air flames at medium and high strain rates remains within the X_O_ = 0.35–0.4 range of oxy-fuel flames. However, at low strain rates, a higher oxygen concentration in the O_2_/CO_2_ mixture is required to match the critical fuel concentration of air flames under the same conditions.

## 3. Materials and Methods

### 3.1. Theoretical Model

**Figure 14:**
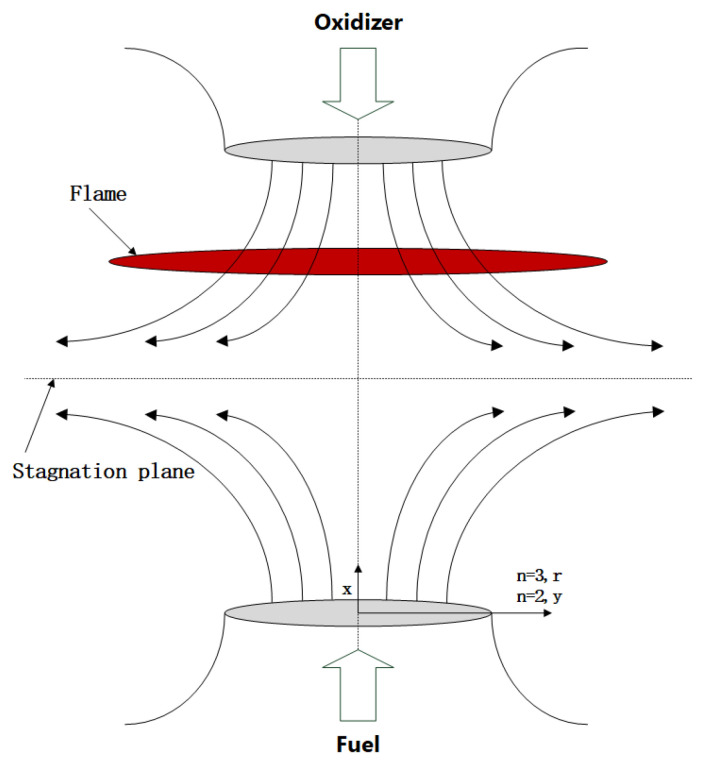
Configuration of the counterflow flame.

The flame configuration investigated in this study is a laminar, axisymmetric counterflow diffusion flame, as illustrated in Figure 14. By assuming a linear variation of radial velocity along the radial direction *r*, the three-dimensional flow analysis can be reduced to a one-dimensional problem. Consequently, both the flame temperature and species mass fractions become functions solely of the axial coordinate *x*. The governing equations are given as follows [37,38]:

(1) $$\mathrm{Continuity}:\;G\left(x\right)=\frac{dF\left(x\right)}{dx}$$

where *x* is the one-dimensional axial spatial coordinate, *G*(*x*) is related to the axial mass flux, and *F*(*x*) is related to the radial mass flux. $G\left(x\right)=-\frac{\rho \upsilon }{r}$, $F\left(x\right)=\frac{\rho u}{2}$, υ is the axial velocity, *u* represents the radial velocity, ρ is the density, and *r* is the one-dimensional radial spatial coordinate.

(2) $$\mathrm{Momentum}:\;H-2\frac{d}{dy}\left(\frac{FG}{\rho }\right)+\frac{3G^{2}}{\rho }+\frac{d}{dy}\left[\mu \frac{d}{dy}\left(\frac{G}{\rho }\right)\right]=0$$

where *H* represents the radial pressure gradient term, $H=\frac{\partial P/\partial r}{r}$, and μ is the mixture dynamic viscosity.

(3) $$\mathrm{Energy}:\;\rho c_{\;P}u\frac{dT}{dx}-\frac{d}{dx}\left(\lambda \frac{dT}{dx}\right)+\rho \sum_{k}c_{Pk}Y_{k}V_{k}\frac{dT}{dx}+\sum_{k}h_{k}{\dot{\omega }}_{k}+Q_{rad}=0$$

where *P* is the pressure, *C_P_* is the specific heat capacity at constant pressure, *C_Pk_* is the specific heat capacity of species *k* at constant pressure, *Y_k_* is the mass fraction of species *k*, *h_k_* is specific enthalpy of species *k*, ${\dot{\omega }}_{k}$ is the molar production rate of species *k*, *Q_rad_* is the radiative heat loss, and *V_k_* is the diffusion velocity of species *k*.

(4) $$\mathrm{Species}:\;\rho u\frac{dY_{k}}{dx}+\frac{d}{dx}\left(\rho Y_{k}V_{k}\right)-{\dot{\omega }}_{k}W_{k}=0,k=1,2\ldots K$$

where *K* is the total number of species and $W_{k}$ is the molar mass of species *k*.

Numerous studies have demonstrated that GRI-Mech 3.0 is well suited for simulating methane–air flames under various conditions [14,39], as well as oxy-fuel flames [27,28,29,40]. Therefore, this mechanism was also adopted in the present work. The model incorporates the Soret effect (thermal diffusion) and a multicomponent transport formulation. The conventional approach for determining extinction limits involves gradually increasing the velocities of both the fuel and oxidizer streams while maintaining equal momentum between the two jets until flame extinction occurs. In contrast, the focus of this study is to identify the minimum methane concentration on the fuel side at a fixed strain rate. To achieve this, the oxidizer flow speed is held constant, while the fuel concentration and fuel stream speed are simultaneously adjusted to conserve momentum. As the flame approaches extinction, the methane concentration on the fuel side is decreased with a step size of 1% for each simulation case.

(5) $$u_{F}=u_{O}\sqrt{\frac{\rho_{O}}{\rho_{F}}}$$

The fuel stream speed is then calculated according to Equation (1). Once the velocities of both the fuel and oxidizer streams are determined, the strain rate of the flame is obtained from Equation (2) [41].

(6) $$a=2\left(u_{O}+u_{F}\sqrt{\frac{\rho_{F}}{\rho_{O}}}\right)/d$$

where u and ρ denote the flow speed and density at the burner outlet, respectively. The subscripts *O* and *F* represent the oxidizer and fuel streams, respectively. The parameter *L* denotes the distance between the fuel and oxidizer nozzles.

This study focuses on investigating the effects of radiation on flame combustion characteristics. Previous research has demonstrated that radiative effects become particularly significant at low strain rates. If the distance between the fuel and the oxidizer nozzle is set too small, the jet velocity profile near the nozzle outlet may not be fully developed, resulting in the flow field not reaching the fully developed, one-dimensional stationary flow assumption. Numerically, this is manifested as non-physical oscillations in the flame temperature profile or drastic and discontinuous changes in the temperature gradient in space [42]. To avoid the boundary effect of flames, in this study, the nozzle distance *L* was set at 10 cm to ensure the formation of a fully developed laminar flow speed profile in the flame residence area. The ambient temperature and pressure were set to 300 K and 1 atm, respectively.

### 3.2. Radiation Models

The governing equations were solved using a modified version of the OPPDIF code for counterflow flames. A radiative source term was incorporated into the energy conservation equation. To quantify radiative heat loss during the combustion process, three distinct approaches were employed in this study: the adiabatic condition (ADI), optically thin (OTM), and statistical narrow-band (SNB) models.

#### 3.2.1. Optically Thin Model (OTM)

The optically thin model (OTM) neglects radiation reabsorption, assuming that all radiative heat emitted by the flame is lost to the surroundings. The flames investigated in this study contain significant amounts of CO_2_ and H_2_O, both of which are strong radiative gases. To ensure computational accuracy, the radiative effect of CO was also considered. The radiative heat loss calculated by the OTM is given by the following expression:

(7) $$q_{rad}=4k_{P}\sigma \left(T^{4}-T_{0}^{4}\right)$$

where $k_{P}$ is the global Planck mean absorption coefficient of the gas mixture, σ is the Stefan–Boltzmann constant, and $T_{0}$ is the ambient temperature at infinity. In $k_{P}=P_{CO_{2}}\alpha_{CO_{2}}+P_{H_{2}O}\alpha_{H_{2}O}+P_{CO}\alpha_{CO}$, $\alpha_{k}$ denotes the Planck mean absorption coefficient of species *k*. In this study, the Planck mean absorption coefficients for CO_2_, CO, and H_2_O were obtained by fitting the data provided by Mitsumasa et al. [43].

By comparing the computational results from the ADI and OTM simulations, the impact of radiative heat loss on flame characteristics could be systematically evaluated.

#### 3.2.2. Statistical Narrow-Band Model (SNB)

In contrast to the OTM, the SNB model accounts for radiation reabsorption effects, making the radiation calculation significantly more complex. This model requires the consideration of detailed spectral structures. The radiative source term depends on the entire temperature and mole fraction fields through absorption, as it derives the gas absorption coefficient from the gas transmissivity.

The SNB model assumes that the spectral radiative flux at any point exclusively depends on the gas absorption coefficient. Furthermore, the integration over the wavelength spectrum can be transformed into an integration over the absorption coefficient domain:

(8) $${\bar{\varphi }}_{\nu }=\frac{1}{\Delta \nu }\int_{\Delta \nu }\varphi \left(\kappa_{\nu }\right)d\nu =\int_{0}^{\infty }g\left(k\right)\varphi \left(k\right)dk=\int_{0}^{1}\varphi \left(g\right)dg=\sum_{i=1}^{N}\omega_{i}\varphi \left(g_{i}\right)$$

where $g\left(k\right)$ is the cumulative distribution function, $g\left(k\right)$ represents the cumulative function, $\omega_{i}$ is the weight function at the Gaussian quadrature point, $g_{i}$ is the corresponding Gaussian point, and $f\left(k\right)dk$ indicates the fraction of the wave number interval from *k* to *k +* Δ*k* occupied by the absorption coefficient.

In the SNB model, the narrow-band average transmissivity can be derived from the distribution function of the narrow-band average absorption coefficient. The distribution function for the narrow-band average absorption is given as follows:

(9) $$f\left(\kappa \right)=\frac{1}{2}\kappa^{-3/2}{\left(BS\right)}^{1/2}\exp\left(\frac{\pi B}{4}\left(2-\frac{\kappa }{S}-\frac{S}{\kappa }\right)\right)$$

The cumulative distribution function of the narrow-band average absorption for an isothermal and non-uniform radiating gas is defined as follows:

(10) $$g\left(\kappa \right)=\int_{0}^{\kappa }f\left(\kappa^{\prime }\right)d\kappa^{\prime }$$

By substituting Equation (9) into Equation (10), a detailed expression for the cumulative distribution function is obtained as follows:

(11) $$g\left(\kappa \right)=\frac{1}{2}\left[1-erf\left(\frac{a}{\sqrt{\kappa }}-b\sqrt{\kappa }\right)\right]+\frac{1}{2}\left[1-erf\left(\frac{a}{\sqrt{\kappa }}+b\sqrt{\kappa }\right)\right]e^{\pi B}$$

where $a=\sqrt{\pi BS}/2$, $b=\sqrt{\pi B/S}/2$. The error function is defined as follows:

(12) $$erf\left(x\right)=\frac{2}{\sqrt{\pi }}\int_{0}^{x}e^{-t^{2}}dt$$

The narrow-band average intensity is calculated using the cumulative distribution function as follows:

(13) $${\bar{I}}^{n}=\frac{1}{\Delta \nu }\int_{\Delta \nu }I_{\nu }d\nu =\int_{0}^{1}I\left(g\right)dg=\sum_{i=1}^{N}w_{i}I\left(g_{i}\right)$$

where $g_{i}$ and $w_{i}$ are the values and weight factors of the Gaussian points in the Gaussian quadrature, respectively.

By incorporating the radiative transfer equation and substituting the radiative flux with spectral radiation intensity for the solution, the radiative source term is obtained as follows:

(14) $$q_{rad}=\sum_{all\Delta \nu }{\bar{I}}_{\nu }\Delta \nu =\sum_{i=1}^{N}\omega_{i}I\left(k_{i}\right)\Delta \nu$$

Further details regarding the SNB radiation model can be found in references [44,45,46,47]. By comparing the computational results of the SNB and OTM models, the influence of radiation reabsorption effects on flame characteristics can be thoroughly discussed.

## 4. Conclusions

This study numerically investigates the effects of radiative heat loss on flame temperature and fuel limits in methane–air and methane oxy-fuel flames under various strain rates using a counterflow diffusion flame model. The influence of radiation reabsorption is specifically evaluated with the statistical narrow-band (SNB) model. The main findings can be summarized as follows:

(a) For methane–air flames, radiative heat loss leads to a radiative extinction limit at low strain rates, reduces the flame temperature, and diminishes the flammable area. Radiation reabsorption only has a minor influence on the fuel limits and flammable area of methane–air flames, primarily at low strain rates. Therefore, the OTM can be reasonably applied to calculate relevant flame characteristics for methane–air flames.
(b) For methane oxy-fuel flames, the influence of radiation reabsorption on flame temperature becomes more pronounced as the strain rate decreases. When calculating the maximum flame temperature as a function of the strain rate, the peak temperature occurs at a lower strain rate in the SNB model compared with the OTM, which neglects reabsorption, indicating that the OTM overestimates radiative heat loss. Radiation reabsorption significantly affects both the critical fuel concentration and the flammable area of methane oxy-fuel flames at low strain rates. The variation of the flammable region with strain rate differs among the three models. For the ADI and SNB models, the flammable area follows S_Low_ > S_Mid_ > S_High_, whereas for the OTM, the trend is S_Mid_ > S_high_ > S_Low_.
(c) By comparing the critical fuel concentrations of air flames and oxy-fuel flames under different radiation models, it is observed that at medium and high strain rates, the (X_F_)_min_ values of air flames fall within the range of X_O_ = 0.35–0.4 for oxy-fuel flames. However, at low strain rates, a higher oxygen concentration is required in the oxy-fuel flame to match the (X_F_)_min_ value of the air flame under the same conditions. Therefore, the SNB model should be used for calculating oxy-fuel flames to ensure higher accuracy.

## Figures and Tables

**Table 1:** Critical fuel concentrations of different radiation models at low, medium and high strain rates.

	a = 10 s^−1^	a = 80 s^−1^	a = 200 s^−1^
ADI	0.12	0.19	0.30
SNB	0.13	0.20	0.31
OTM	0.14	0.20	0.31

**Table 2:** The critical fuel concentrations of different strain rates under the ADI, OTM, and SNB models at different oxygen concentrations.

Model	a (s^−1^)	X_O_ = 1.0	X_O_ = 0.8	X_O_ = 0.6	X_O_ = 0.4	X_O_ = 0.35	X_O_ = 0.30
ADI	10	0.09	0.10	0.11	0.13	0.14	0.17
	80	0.11	0.12	0.14	0.18	0.22	0.3
	200	0.13	0.14	0.16	0.22	0.3	0.49
SNB	10	0.11	0.13	0.14	0.16	0.19	0.26
	80	0.12	0.13	0.15	0.20	0.23	0.35
	200	0.13	0.14	0.15	0.23	0.31	0.54
OTM	10	0.14	0.16	0.18	0.29	0.35	0.55
	80	0.12	0.13	0.17	0.21	0.25	0.38
	200	0.13	0.14	0.20	0.25	0.32	0.57

**Table 3:** The critical oxygen concentrations of ADI, OTM and SNB models at X_F_ = 1 and different strain rates.

	ADI	SNB	OTM
a = 10 s^−1^	0.22	0.23	0.27
a = 80 s^−1^	0.25	0.25	0.26
a = 200 s^−1^	0.28	0.28	0.29

## Data Availability

The original contributions presented in this study are included in the article. Further inquiries can be directed to the corresponding author.
